# Possible Role of Interleukin-31/33 Axis in Imatinib Mesylate-Associated Skin Toxicity

**DOI:** 10.4274/tjh.2014.0021

**Published:** 2015-05-08

**Authors:** Caterina Musolino, Alessandro Allegra, Carmen Mannucci, Sabina Russo, Andrea Alonci, Valerio Maisano, Gioacchino Calapai, Sebastiano Gangemi

**Affiliations:** 1 University of Messina Faculty of Medicine, Department of General Surgery and Oncology, Division of Hematology, Messina, Italy; 2 Azienda Ospedaliera Universitaria Policlinico “G. Martino”, Department of Clinical and Experimental Medicine, Operative Unit of Clinical Pharmacology, Messina, Italy; 3 University of Messina Faculty of Medicine, Policlinic “G. Martino”, Department of Clinical and Experimental Medicine, Division of Allergy and Clinical Immunology, Messina, Italy; 4 IFC CNR, Messina Unit, Institute of Clinical Physiology, Messina, Italy

**Keywords:** Interleukin-31 (IL-31), Interleukin-33 (IL-33), Tyrosine kinase inhibitors, Imatinib mesylate, Chronic myeloid leukemia, Pruritus

## Abstract

Imatinib mesylate is a small-molecule tyrosine kinase inhibitor (TKi) designed to target c-ABL and BCR-ABL, approved for the treatment of chronic myeloid leukemia and gastrointestinal stromal tumors. Adverse cutaneous reactions induced by imatinib are frequent, generally moderate, and dose-dependent. The aim of this work was to investigate the possible contribution of interleukin (IL)-33 and IL-31, cytokines involved in disorders associated with itching, in the pathogenesis of pruritus in a patient undergoing imatinib mesylate treatment. His IL-31 and IL-33 serum levels were significantly higher than in the control group (respectively 96.6 pg/mL vs. 7.623±7.681 pg/mL and 27.566 pg/mL vs. 6.170±7.060 pg/mL). In light of these findings, imatinib mesylate-related symptoms of dermatologic toxicities might be related to the release of IL-31 and IL-33. In particular, it is supposable that TKi usage could cause keratinocyte injury, the release of IL-33, and the consequent interaction with its receptor on mast cells that induces the secretion of several factors capable of causing skin manifestations, including IL-31, a known pruritus-inducing cytokine. This report, to the best of our knowledge, is the first work describing the possible involvement of the IL-31/IL-33 axis in the pathogenesis of skin side effects related to imatinib mesylate treatment.

## Abstract (tr)

İmatinib mesilat c-ABL ve BCR-ABL’yi hedeflemek için tasarlanan küçük- molekül tirozin kinaz inhibitörü (TKİ) olup kronik miyeloid lösemi ve gastrointestinal stromal tümör tedavisi için onaylanmıştır. İmatinib ile indüklenen advers kutanöz reaksiyonlar nadir, genellikle ılımlı ve doz bağımlıdır. Bu çalışmanın amacı, kaşıntı ile ilişkili bozukluklar ile ilgili sitokinler olan interlökin (IL)-33 ve IL-31’in imatinib mesilat tedavisi alan bir hastada kaşıntı patogenezine olası katkısını araştırmak idi. Hastanın serum IL-31 ve IL-33 düzeyleri kontrol grubundan anlamlı olarak yüksek idi (sırasıyla; 96,6 pg/mL vs. 7,623±7,681 pg/mL ve 27,566 pg/mL vs. 6,170±7,060 pg/mL). Bu bulgular ışığında, imatinib mesilat- ile ilişkili dermatolojik toksisiteler IL-31 ve IL-33 salınımı ile ilişkili olabilir. Özellikle, TKİ kullanımının keratinosit hasarı, IL-33 salınımı ve mast hücre yüzeyindeki reseptörü ile karşılıklı etkileşimi sonucu, deri bulgularına yol açma yeteneği olan, kaşıntıyı-indükleyen bir sitokin olarak bilinen IL-31’de içeren çeşitli faktörlerin sekresyonuna sebep olabileceği varsayılabilir. Bu rapor, bildiğimizce, imatinin mesilat tedavisi ile ilişkili deri yan etkilerinin patogenezinde interlökin-31/33 aksının olası rolünü tanımlayan ilk çalışmadır.

## INTRODUCTION

Imatinib mesylate is a tyrosine kinase inhibitor (TKi) approved for the treatment of chronic myeloid leukemia (CML) and gastrointestinal stromal tumors [1]. Several case reports noted the occurrence of dose-limiting skin disorders during imatinib administration [2,3,4,5,6]. Interleukin (IL)-33 is a recently recognized cytokine that appears to drive T helper type 2 (Th2) responses [7,8,9]. IL-33 has been linked to important diseases, including asthma, rheumatoid arthritis, ulcerative colitis, and metabolic, neurologic, and cardiovascular diseases.

IL-31 is a member of the IL-6 family of cytokines, mainly expressed in pruritic disorders [10]. IL-31 is a Th2 cytokine that is mainly produced by the CD45RO+ cutaneous lymphocyte antigen-positive T lymphocytes. It is involved in both innate and adaptive immunity in tissues that are in close contact with the external environment, i.e. the skin [11]. Recently IL-31 has been demonstrated to be produced by human mast cells [11]; in addition, monocytes, macrophages, and monocyte-derived dendritic cells produce IL-31. Moreover, epidermal keratinocytes and dermal fibroblasts show enhanced IL-31 mRNA expression upon H2O2 stimulation [10]. Enhanced expression of IL-31 is associated with a number of diseases, including pruritic diseases such as atopic dermatitis, allergic contact dermatitis, prurigo nodularis, and chronic urticaria [12].

In a previous work we reported a significant increase of IL-31 and IL-33 serum levels in a patient with a bronchoalveolar carcinoma, who had shown previous skin rash, xerosis, and pruritus during treatment with different EGFR-TK inhibitors [13]. The aim of this work was to investigate the possible contribution of IL-31 and IL-33, cytokines involved in disorders associated with itching, in the pathogenesis of pruritus in a patient undergoing imatinib mesylate treatment.

## CASE PRESENTATION

A 73-year-old man, while being evaluated for splenomegaly, showed leukocytosis upon peripheral blood examination with low hemoglobin and normal platelet count. His past medical history included hypertension, stroke, and dyslipidemia. He had no history of drug allergy.

His provisional diagnosis was CML, which was subsequently confirmed by the presence of Philadelphia (Ph) chromosome [Ph+ t (9;22) (q34;q11)] in 100% of the cells in metaphase. He was started on cytoreduction with hydroxyurea. Subsequently, he started to take imatinib mesylate at 400 mg once daily. While on therapy, he developed pruritus. Physical examination revealed erythema of the skin associated with mild exfoliation, which affected mainly the upper and lower limbs. There was no history of application of or contact with any irritant substances. Systemic antihistamines were administered. Moreover, the patient was treated with a short course of corticosteroids along with topical clobetasol propionate. Imatinib mesylate was discontinued for 2 weeks, and the patient showed some improvement. Imatinib was restarted at 100 mg once daily and was gradually built up to 300 mg once daily with reappearance of the pruritus. 

On further follow-up, he had achieved complete hematologic response at 6 months, but failed to achieve a cytogenetic response or a major molecular response at 12 months. His pruritus has become constant and involves his entire body. He is unable to sleep unless medicated with sedatives. The patient is still only in complete hematological response and our intent is to shift to a second-line TKi. 

We evaluated IL-31 and IL-33 serum levels in this patient and in 18 sex- and age-matched healthy controls. The study was conducted according to good clinical and laboratory practice rules and the principles of the Declaration of Helsinki, and it was approved by the local ethics committee. After obtaining written informed consent, blood samples were collected to determine IL-31 and IL-33 serum levels. 

We used a standard sandwich ELISA kit (USCN Life Science, Houston, TX, USA). The lower limit of detection was determined as suggested by the manufacturer, as follows: (mean negative control optical density) + 2 x (StDev of negative control optical density). The absorbance was measured at 450 nm. 

The patient’s IL-31 and IL-33 serum levels were markedly higher than those in the control group (respectively 96.6 pg/mL vs. 7.623±7.681 pg/mL and 27.566 pg/mL vs. 6.170±7.060 pg/mL).

## DISCUSSION AND REVIEW OF THE LITERATURE

Imatinib mesylate is a small-molecule TKi designed to target c-ABL and BCR-ABL, but it is also able to target KIT and the platelet-derived growth factor (PDGF) receptor.

Adverse cutaneous reactions induced by imatinib are frequent, generally moderate, and dose-dependent [14,15], although all grades of cutaneous reactions have been reported, ranging from exfoliative dermatitis to vesicular rash and Stevens-Johnson syndrome [16,17,18,19].

Concerning the pathogenesis of skin reactions occurring during imatinib administration, a direct effect of the tyrosine kinase inhibition on the PDGF receptor, expressed on dermal mast cells and blood vessels, was suggested [20]. 

The inhibition of this receptor might cause an augmentation of dermal interstitial fluid pressure with subsequent phenomena of skin edema and erythema. However, the histological evidence for an augmented number of dermal mast cells, which express a functional c-kit receptor, in cases of severe skin toxicity from imatinib mesylate seems to exclude a direct effect of the drug on mast cells themselves [21,22].

As a result, it has also been proposed that imatinib mesylate might operate as a dose-dependent inducer of chemoattractant substances able to induce pruritus [21], such as IL-33 and IL-31. 

IL-33 has recently been attributed to the epithelial ‘alarmin’ defense system. IL-33 is liberated by the epithelial cells in several tissues and organs, including keratinocytes, immune cells, and endothelial cells [9,23]. 

It has been proven that IL-33 is recognized by T1/ST2 receptors on the surface of mast cells; this results in the secretion of proinflammatory factors, including IL-6, TNF-α, and leukotrienes. Subsequently, these signals can cause changes, including vasodilatation, increased permeability of the microvasculature, and recruitment of inflammatory cells [23].

The link between pruritus and IL-31 has also been confirmed by a study showing that transgenic mice models over-expressing IL-31 developed severe pruritus and an increase in mast cells [24]. Moreover, it is probable that IL-31 may generate pruritus through the induction of a yet unknown keratinocyte-derived mediator, which subsequently activates unmyelinated C fibers in the skin [25].

It is presumable that the skin manifestations and itch caused by imatinib mesylate treatment could be related to the release of IL-31 and IL-33. It is supposable that TKi usage can cause keratinocyte injury with the release of IL-33, which in turn interacts with its receptor on mast cells, leading to the secretion of several factors capable of causing skin manifestations, including IL-31 [26,27].

Finally, our finding of very high serum levels of IL-33 and IL-31 in a CML patient undergoing imatinib mesylate treatment compared to healthy controls has a more relevant significance in light of previous works, where we found a significant decrease of IL-33 plasma levels in patients affected by myeloproliferative disorders such as polycythemia vera and essential thrombocythemia and in subjects with other hematologic diseases [28,29,30]. For this reason, although we have no information about basal IL-31 and -33 levels in our CML patient before imatinib treatment, we think that the increase of the values of cytokines after imatinib treatment with respect to the controls is significant.

In conclusion, although our report, with the description of a unique case, does not permit us to draw sure conclusions on the possible association between itch and TKi usage, further studies conducted using different TKis such as nilotinib and dasatinib will be useful to better define the role of these cytokines in these patients.
